# Assessing arterial stiffness using characteristics of Korotkoff sounds

**DOI:** 10.3389/fcvm.2026.1654162

**Published:** 2026-02-10

**Authors:** Shuqi Ren, Wei Zhao, Changcheng Yi, Xiaoyan Deng, Zengsheng Chen, Li Wang, Ling Xu, Yuheng Yang, Yubo Fan, Anqiang Sun

**Affiliations:** 1 Key Laboratory of Biomechanics and Mechanobiology (Ministry of Education), Key Laboratory of Innovation and Transformation of Advanced Medical Devices (Ministry of Industry and Information Technology), National Medical Innovation Platform for Industry-Education Integration in Advanced Medical Devices (Interdiscipline of Medicine and Engineering), School of Biological Science and Medical Engineering, Beihang University, Beijing, China; 2 Department of Cardiology, Institute of Vascular Medicine, State Key Laboratory of Vascular Homeostasis and Remodeling, NHC Key Laboratory of Cardiovascular Molecular Biology and Regulatory Peptides, Beijing Key Laboratory of Cardiovascular Receptors Research, Peking University Third Hospital, Peking University, Beijing, China; 3 Physical Examination Center, Peking University Third Hospital, Beijing, China

**Keywords:** arterial stiffness, artificial intelligence, blood pressure, cardiovascular disease, machine learning, pulse wave velocity

## Abstract

**Background and Objectives:**

Arterial stiffness is a recognized marker of vascular ageing and is associated with adverse cardiovascular outcomes. However, routine assessment of pulse wave velocity (PWV) remains limited in many clinical and home settings. This study investigated the feasibility of extracting arterial stiffness-related information from Korotkoff sounds recorded during cuff-based blood pressure measurement using feature analysis and machine learning.

**Materials and methods:**

Korotkoff sounds were collected from 123 young (25.9 ± 2.2 years) participants and 112 older (67.5 ± 6.7 years) participants using a custom-developed device as a proof-of-concept for age-related vascular differences. In addition, 81 hospital participants with measured brachial-ankle PWV (baPWV) were enrolled and grouped according to baPWV to further evaluate clinical feasibility. Time- and frequency-domain features were extracted, and both traditional feature-based models and deep learning approaches were applied for classification.

**Results:**

Extracted features including center of mass, skewness, and peak frequency showed significant differences between the age-stratified groups. Two deep learning models achieved classification accuracies of 89.3% and 93.7%, respectively, outperforming traditional feature-based analysis. In the baPWV-defined classification task, model performance was moderate (accuracy 87.5% and 81.3%). In the baPWV-measured cohort, Korotkoff sound-derived features showed a statistically significant but modest association with measured baPWV.

**Conclusion:**

Korotkoff sounds contain measurable information related to vascular ageing and arterial stiffness, and machine learning can leverage these signals for group discrimination. Given that the primary comparison used age as a surrogate label and clinical outcomes were not assessed, the present data do not establish incremental value for cardiovascular risk stratification beyond age and blood pressure. Larger studies with standardized PWV measurements, ideally carotid-femoral PWV (cfPWV), and prospective validation are required before prognostic or risk-stratification claims can be made.

## Introduction

As the ageing population increases globally, the prevention of cardiovascular disease (CVD) faces more severe challenges (1). By 2030, about 20% of the population will be aged 65 years or older, and in this age group, CVD will be the leading cause of death, accounting for 40% of deaths (2). Arterial stiffening is a key manifestation of vascular ageing and is associated with higher cardiovascular morbidity and mortality (3–9). Accordingly, assessment of arterial stiffness has attracted growing interest as a means to characterize vascular health and potentially refine prevention strategies (10–12).

Pulse wave velocity (PWV) is widely used for non-invasive assessment of arterial stiffness (13–16). Importantly, carotid-femoral PWV (cfPWV), which reflects aortic (large elastic artery) stiffness, is regarded as the reference-standard measure for arterial stiffness assessment in clinical practice and research, and standardization of its measurement has been emphasized in expert consensus documents (17). However, cfPWV measurement often requires dedicated equipment and trained operators, which limits access in routine primary care and home settings. Recent hypertension guidelines underscore the need for pragmatic approaches that can be implemented broadly across care settings (18).

In contrast to dedicated PWV devices, Korotkoff sounds are already routinely generated during cuff-based blood pressure measurement. Although their precise genesis remains debated, both arterial wall mechanics (compliance-related vibration) (19, 20) and hemodynamic factors (flow patterns) (21–23) are believed to contribute, suggesting that Korotkoff sounds may contain information related to vascular properties. Evidence in recent years (24–26) has increasingly supported the concept that Korotkoff sounds encode physiologically meaningful signatures beyond systolic/diastolic pressure, motivating efforts to extract vascular information from these signals.

Korotkoff sounds are complex, non-stationary biosignals, posing analytical challenges similar to heart sounds and other physiological waveforms. Conventional approaches typically rely on engineered time- and frequency-domain features, whereas deep learning can learn high-dimensional representations directly from time–frequency maps. Transfer learning with convolutional neural networks (CNNs) has proven effective in biosignal classification when domain-specific labeled datasets are limited (27–30).

In this study, we investigated whether machine-learning analysis of Korotkoff sounds can capture stiffness-related information. We performed (i) a proof-of-concept analysis comparing young and older adults (age-defined groups expected to differ in vascular ageing), and (ii) an additional clinical feasibility analysis in a separate cohort with measured PWV, to examine whether Korotkoff sound–derived features and models relate to PWV. Because age itself (and estimated PWV derived from age and blood pressure) can be used for risk estimation, the present work focuses on feasibility rather than establishing incremental prognostic value beyond conventional variables (31).

## Methods

**Figure 1:**
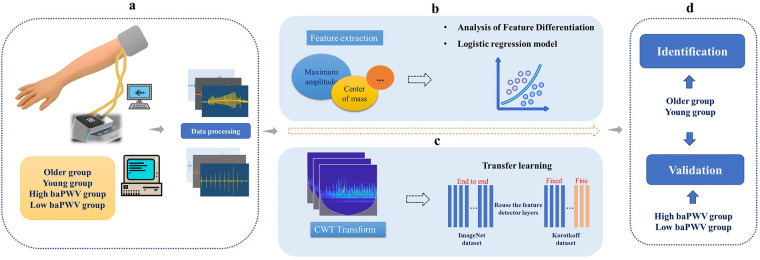
Schematic diagram of the study methodology. baPWV, brachial-ankle pulse wave velocity. CWT, continuous wavelet transforms. **(a)** Data Acquisition and Preprocessing: Korotkoff sound signals were collected and preprocessed. Participants were stratified into older group vs. young group (age-stratified), and into high-baPWV group vs. low-baPWV group. **(b)** Traditional Feature Analysis: Time- and frequency-domain features were extracted from the signals and compared between the older group and young group to identify significant differences. A logistic regression model was subsequently developed for classification. **(c)** Deep Learning-Based Classification: Time-frequency images were generated via CWT, and convolutional neural networks were trained using a transfer learning approach. **(d)** Clinical Feasibility: The Korotkoff sound–based approach was further evaluated against measured baPWV data.

The overall methodology of this study is outlined in Figure 1. In brief, Korotkoff sound signals were acquired from participants using a self-developed device. Participants were categorized for two complementary analyses: (i) an age-stratified proof-of-concept comparison between an older group and a young group (age used as a surrogate of vascular ageing rather than a direct stiffness diagnosis), and (ii) in a separate clinical feasibility cohort with measured brachial-ankle pulse wave velocity (baPWV), a comparison between high-baPWV and low-baPWV groups based on measured baPWV values. Furthermore, the correlation between baPWV and the acoustic features was investigated to explore clinical relevance of the proposed method.

### Hardware device

**Figure 2:**
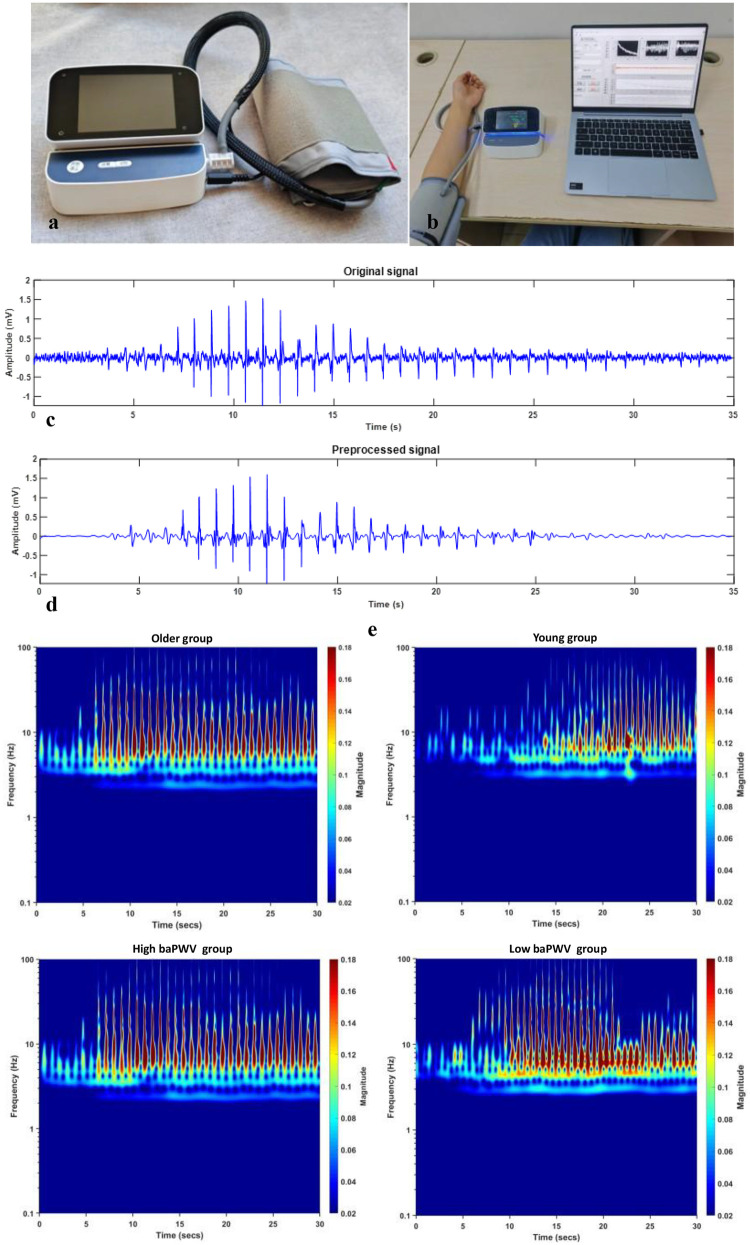
baPWV, brachial-ankle pulse wave velocity. **(a)** The self-developed Korotkoff sound signal acquisition device. **(b)** The process of collecting signals. **(c)** The original Korotkoff sound signal. **(d)** The preprocessed Korotkoff sound signal. **(e)** The time-frequency RGB images after continuous wavelet transform (older group vs. young group, high-baPWV group vs. low-baPWV group).

Signal acquisition was performed using a custom-developed Korotkoff sound acquisition device (Figure 2A), which comprised three primary components: a hardware unit, an inflatable cuff, and a sensor integrated into the cuff for Korotkoff sound detection. The hardware unit contained a pump for cuff inflation and deflation, an STM32H750 microprocessor with 24-bit AD sampling for data acquisition and system control, and a Bluetooth module for communication with a computer. To balance performance and cost, a CM-01B sensor was utilized for collecting Korotkoff sound signals (32), while an MPS20N0040D-S sensor was employed to acquire the cuff pressure signal. All Korotkoff sound signals were sampled at a frequency of 1,000 Hz.

In the clinical feasibility cohort, baPWV was measured as the comparator PWV index using an automatic waveform analyzer (BP-203 RPE III, Omron Health Medical, Dalian, China). baPWV was used as the comparator PWV measure because it is widely implemented in clinical workflows and population studies and can be obtained with high operational efficiency (33–36).

### Data acquisition

Arterial structure and function change with age, resulting in progressive arterial stiffness (13). This well-established relationship, demonstrated by Gomez-Sanchez et al. in a Spanish adult cohort without overt CVD (10), provided the rationale for using age as a primary grouping variable in the proof-of-concept analysis. An *a priori* power analysis was conducted to ensure sufficient statistical power, defined as an 80% probability of detecting a true effect at a significance level (α) of 0.05. Based on a reference effect size from a similar study (26), the total required sample size was estimated to be 200. Therefore, we collected Korotkoff sound signals from 112 older adults (67.5 ± 6.7 years) and 123 young adults (25.9 ± 2.2 years), as the older and young groups, respectively. To evaluate clinical feasibility using a measured arterial stiffness index, Korotkoff sound and concurrent baPWV data were additionally collected from 81 hospital participants. Based on the median baPWV value of this cohort, participants were stratified into two groups (high baPWV vs. low baPWV) for PWV-defined classification and association analyses.

Participants for the primary comparison (older vs. young groups) were recruited from the community. Korotkoff sound recordings were obtained with the participant seated and the test arm supported such that the cuff on the mid-upper arm was positioned at heart level. The cuff was applied directly on the bare upper arm (participants removed thick clothing from the measurement arm) to minimize signal attenuation and artifacts. The sensor was positioned over the brachial artery pulse point. Following cuff inflation to achieve complete arterial occlusion, signals were acquired during a controlled deflation period. All participants provided written informed consent and completed a questionnaire covering demographics (age, sex, height, weight), medication use, medical history, and smoking status. Blood pressure and heart rate were obtained during the study visit using standardized measurements performed by trained staff and were recorded alongside the Korotkoff sound acquisition. The inclusion criteria for the young and older groups were: age 20–30 or 60–79 years, respectively; a body mass index (BMI) of 20–25 kg/m²; good general health without a history of major diseases; non-smoking status; and normal blood pressure and heart rate. Representative Korotkoff sound recordings from older and young subjects are provided in the Supplementary Material Audio.

The data for the high-baPWV and low-baPWV groups were collected at a hospital. All the participants provided informed consent and completed a detailed questionnaire (as described above). Subjects aged 20–79 years with no serious diseases (including cardiovascular diseases, malignant tumors, severe primary liver or kidney diseases, or chronic lung diseases) were included. Participant characteristics are summarized in Table 1 (age-stratified cohort) and Table 2 (baPWV-measured hospital cohort).

**Table 1:** Statistical information for older and young study participants.

Variables	Older (*n* = 112)	Young (*n* = 123)	*P*
Age in years, M (SD)	67.5 (6.7)	25.9 (2.2)	<0.001
Gender, *n* (%)			0.112
Male	44 (39.4)	57 (46.3)	
Female	68 (60.7)	66 (53.7)	
BMI, M(SD)	23.4 (1.5)	22.8 (1.1)	0.115
Blood pressure, M(SD)			
SBP	120 (11)	118 (6)	0.083
DBP	79 (6)	77 (5)	0.091
Heart rate, M (SD)	78.2 (11.4)	76.0 (9.2)	0.129

BMI, body mass index; SBP, systolic blood pressure; DBP, diastolic blood pressure; M, mean; SD, standard deviation. Participants were categorized into the older group and young group based on age as a well-established surrogate for arterial stiffness. Data are presented as mean (SD) for continuous variables and number (percentage) for categorical variables. Group differences were assessed using independent samples *t*-tests for continuous variables and chi-square tests for categorical variables. A *p*-value < 0.05 was considered statistically significant. All participants in the age-stratified community cohort were non-smokers and reported no major medical history per inclusion criteria.

**Table 2:** Statistical information for study participants with brachial-ankle pulse wave velocity data included.

Variables	High baPWV (*n* = 45)	Low baPWV (*n* = 36)
Age in years, M (SD)	51.8 (14.2)	36.7 (9.7)
Gender, *n* (%)		
Male	25 (55.6)	11 (30.6)
Female	20 (44.4)	25 (69.4)
BMI, M(SD)	26.0 (4.1)	23.0 (3.4)
Blood pressure, M(SD)		
SBP	136 (13)	118 (12)
DBP	81 (9)	69 (11)
Heart rate, M (SD)	74.2 (10.6)	72.0 (11.5)
Diabetes, *n* (%)	8 (17.8)	7 (19.4)
Smoking, *n* (%)	8 (17.8)	3 (8.3)
Past medical history, *n* (%)	27 (60.0)	7 (19.4)

baPWV, brachial-ankle pulse wave velocity; BMI, body mass index; SBP, systolic blood pressure; DBP, diastolic blood pressure; M, mean; SD, standard deviation. Participants were stratified into high-baPWV and low-baPWV groups. Data are presented as mean (SD) for continuous variables and number (percentage) for categorical variables. Past medical history” refers to non-severe chronic conditions (e.g., hypertension, dyslipidemia) and does not include the excluded serious diseases.

### Signal processing and feature extraction

All data processing was performed using MATLAB (version 2019a). The acquired Korotkoff sound signals were first processed to eliminate baseline drift using filtering techniques. Subsequently, high-frequency noise was attenuated employing wavelet transform, which offers superior time-frequency localization for non-stationary signals. Figures 2c,d display representative original and preprocessed Korotkoff sound signals, respectively.

For the preprocessed signals, we introduced the concept of “center of mass” to integrate amplitude and temporal location information. A set of six time-domain features was extracted, including maximum amplitude, kurtosis, skewness, peak factor, pulse factor, and form factor. Additionally, three frequency-domain features were obtained: peak frequency, center of gravity frequency, and mean square frequency. Detailed mathematical descriptions of the preprocessing steps and feature extraction formulas are provided in the Supplementary Material.

### Statistical analysis

All statistical analyses were performed using SPSS (version 27, IBM Corp.). Normality of continuous variables was assessed using the Kolmogorov–Smirnov test. For comparisons of acoustic features between groups, independent-samples *t*-tests were used to compare mean values. Homogeneity of variance was evaluated using Levene's test; when variances were unequal, Welch's *t*-test was applied. To account for multiple comparisons across the ten extracted features, statistical significance for the univariate feature comparisons was set at *p* < 0.005 (Bonferroni correction).

To address potential confounding factors in the correlation analyses between Korotkoff sound features and arterial stiffness (as measured by baPWV), multivariable linear regression analyses were conducted. Candidate covariates for adjustment were pre-specified based on prior literature and clinical plausibility, and their distributions and intercorrelations were also examined in the current dataset. Age, diabetes, obesity (BMI) and systolic blood pressure (SBP) were included as established determinants of baPWV (37–40). Separate regression models were built for each Korotkoff sound feature that demonstrated a significant univariate association with baPWV. In each model, baPWV served as the dependent variable, while the Korotkoff sound feature and covariates (age, diabetes, BMI, SBP) were entered as independent variables. Variance inflation factors (VIFs) were examined to assess multicollinearity (all VIF < 5).

### Machine learning methods

Time-frequency images were obtained using a continuous wavelet transform (CWT). These representations were utilized as input for two CNN models, GoogLeNet and SqueezeNet, which were trained following a transfer learning paradigm to obtain stiffness-related information. A comprehensive description of the machine learning hyperparameters is available in the Supplementary Material.

## Results

### Participant characteristics

#### Time- and frequency-domain features

The statistical results for the ten extracted features are summarized in Table 3. No significant difference was observed in the maximum amplitude value between the older and young groups. This lack of difference may be attributed to the sensitivity of the maximum amplitude to factors such as sensor placement, variations in tissue layer thickness and density, and individual differences in blood flow. Although the sensor is aligned with the relative position of the brachial artery during signal acquisition, it is inevitably influenced by the properties of the lateral tissue of the brachial artery and differences in blood flow through the brachial artery. In contrast, the proposed center of mass feature demonstrated a statistically significant difference between the two groups. This finding suggests that while individual anatomical and physiological factors may influence absolute amplitude values, they exert a comparatively minor effect on the temporal location of the maximum amplitude, supporting the robustness of this feature.

**Table 3:** Statistical results of older and young groups.

Indicates	Young	Older	*P*	T	Cohen's d
Maximum amplitude	1.095 (0.446)	1.059 (0.555)	0.679	0.414	0.072
Kurtosis	14.199 (5.364)	17.094 (4.202)	0.070	−1.828	−0.316
Skewness	0.231 (0.689)	−0.802 (1.371)	<0.001	5.632	0.973
Peak factor	6.941 (1.504)	6.526 (2.421)	0.228	1.211	0.209
Pulse factor	12.860 (3.511)	12.978 (6.127)	0.298	1.045	0.181
Form factor	1.726 (0.238)	1.777 (0.305)	0.279	−1.087	−0.188
Center of mass	0.971 (1.605)	−0.643 (1.574)	<0.001	5.785	0.999
Peak frequency	3.707 (1.478)	2.653 (0.441)	<0.001	5.411	0.935
Center of gravity frequency	28.330 (5.798)	32.417 (13.206)	0.018	−2.388	−0.412
Mean square frequency	7.771 (1.596)	9.514 (5.595)	0.017	−2.427	−0.419

Values are presented as mean (SD). Group differences were assessed using independent-samples *t*-tests (Welch's correction applied when variances were unequal). Cohen's d is provided as a standardized effect size. To account for multiple comparisons across 10 features, statistical significance was set at *p* < 0.005 (Bonferroni correction).

In addition, skewness differed significantly between groups (Table 3), suggesting group-level differences in the Korotkoff sound envelope: the envelope distribution was left-skewed in older adults and right-skewed in young adults. Peak-frequency–related results suggested that spectral energy was concentrated at different frequency ranges between groups, with peak frequency showing a significant shift (Table 3). Center of gravity frequency and mean-square frequency showed nominal between-group differences (*p* < 0.05) but did not remain significant after Bonferroni correction.

All the features were entered into an exploratory logistic regression model. The model demonstrated a good fit per the Hosmer-Lemeshow test (*p* > 0.05) and achieved a classification accuracy of 83.7%. The specific information is shown in Table 4.

**Table 4:** Logistic regression analysis results.

Features	Coef.	SE	Wald *χ*^2^	*P*	OR	95% CI
Maximum amplitude	1.399	0.648	4.663	0.031	4.050	[1.138,14.412]
Kurtosis	0.050	0.097	0.264	0.608	1.051	[0.869,1.272]
Skewness	−1.543	0.521	8.768	0.003	0.214	[0.077,0.594]
Peak factor	1.051	0.632	2.768	0.096	2.861	[0.829,9.873]
Pulse factor	−0.528	0.294	3.212	0.073	0.590	[0.331,1.051]
Form factor	0.333	1.578	0.044	0.833	1.395	[0.063,30.735]
Center of mass	−0.879	0.237	13.768	<0.001	0.415	[0.261,0.660]
Peak frequency	−1.516	0.495	9.372	0.002	0.220	[0.083,0.580]
Center of gravity frequency	−0.041	0.048	0.707	0.400	0.960	[0.874,1.055]
Mean square frequency	0.135	0.201	0.455	0.500	1.145	[0.773,1.697]
Hosmer-Lemeshow test			χ²		*P*	
			6.601		0.580	
Accuracy			83.7%			

Coef., coefficient; SE, standard error; OR, odds ratio; CI, confidence interval. Binary logistic regression was performed to predict the likelihood of being in the older group (coded as 1) vs. the young group (coded as 0). The Hosmer-Lemeshow test yielded a χ² statistic of 6.601 (*p* = 0.580), indicating a good model fit. An OR > 1 indicates higher odds of being classified into the older group (coded as 1), whereas an OR < 1 indicates lower odds.

Although the conventional feature-based analysis demonstrated some ability to distinguish the age-defined groups/stiffness-related signatures, its classification accuracy remained limited. A principal challenge lies in the manual curation of the most discriminative features or identifying optimal multi-dimensional feature combinations to enhance the model's capability for assessing pathological signals. In contrast, deep learning networks possess a superior ability to automatically learn complex, high-dimensional feature hierarchies and approximate the optimal mapping function through iterative parameter optimization. To enable more automated and scalable screening of vascular stiffness-related signatures, we therefore developed and implemented deep learning models.

#### Verification

To evaluate clinical relevance, correlations between Korotkoff sound features and baPWV were analyzed (Table 5). Notably, several features (including center of mass, peak frequency, and skewness) showed modest correlations with measured baPWV, supporting the hypothesis that these acoustic characteristics are sensitive to vascular property differences. Nevertheless, the correlation coefficients were modest, indicating limited effect size; thus, these findings should be interpreted as preliminary evidence requiring replication in larger and independent cohorts.

**Table 5:** Statistical analysis of the correlation between the characteristics of the korotkoff sound signals and the baPWV values of 81 subjects.

Indicates	*P*	r	1-*β*
Maximum amplitude	0.049	0.218	0.228
Kurtosis	0.182	0.103	0.205
Skewness	0.031	0.181	0.233
Peak factor	0.745	0.037	0.056
Pulse factor	0.442	−0.087	0.084
Form factor	0.048	−0.240	0.326
Center of mass	0.005	−0.310	0.506
Peak frequency	0.011	−0.254	0.360
Center of gravity frequency	0.195	0.146	0.148
Mean square frequency	0.847	−0.022	0.052

baPWV, brachial-ankle pulse wave velocity; P, significance level; r, correlation coefficient; 1-β, statistical power. The correlations between features of Korotkoff sound signals and baPWV values were assessed using Pearson's correlation analysis. The correlation coefficient (r) indicates the strength and direction of the linear relationship, where values around 0.1, 0.3, and 0.5 are typically considered small, medium, and large effects, respectively. Statistical power (1-β) represents the probability of correctly rejecting a false null hypothesis, with values above 0.8 considered desirable. The modest correlation coefficients and variable statistical power observed here suggest that these preliminary findings should be validated in larger-scale studies.

To assess whether Korotkoff sound features provide information beyond established covariates, multivariable linear regression analyses were performed adjusting for age, diabetes status, BMI, and systolic blood pressure. After adjustment, skewness (*β* = 0.166, *p* = 0.030), center of mass (*β* = −0.310, *p* = 0.018), and peak frequency (*β* = −0.331, *p* = 0.029) remained significantly associated with baPWV. The negative coefficients indicate that higher values of center of mass and peak frequency are associated with lower baPWV (i.e., less stiffness), consistent with the between-group differences observed in the age-defined task. The relatively high adjusted R² values primarily reflect the contribution of established covariates (especially age and blood pressure), while the persistence of the acoustic features suggests a small independent association. Full model details are provided in the Supplementary Material Tables S3–S5.

#### CNN models

**Figure 3:**
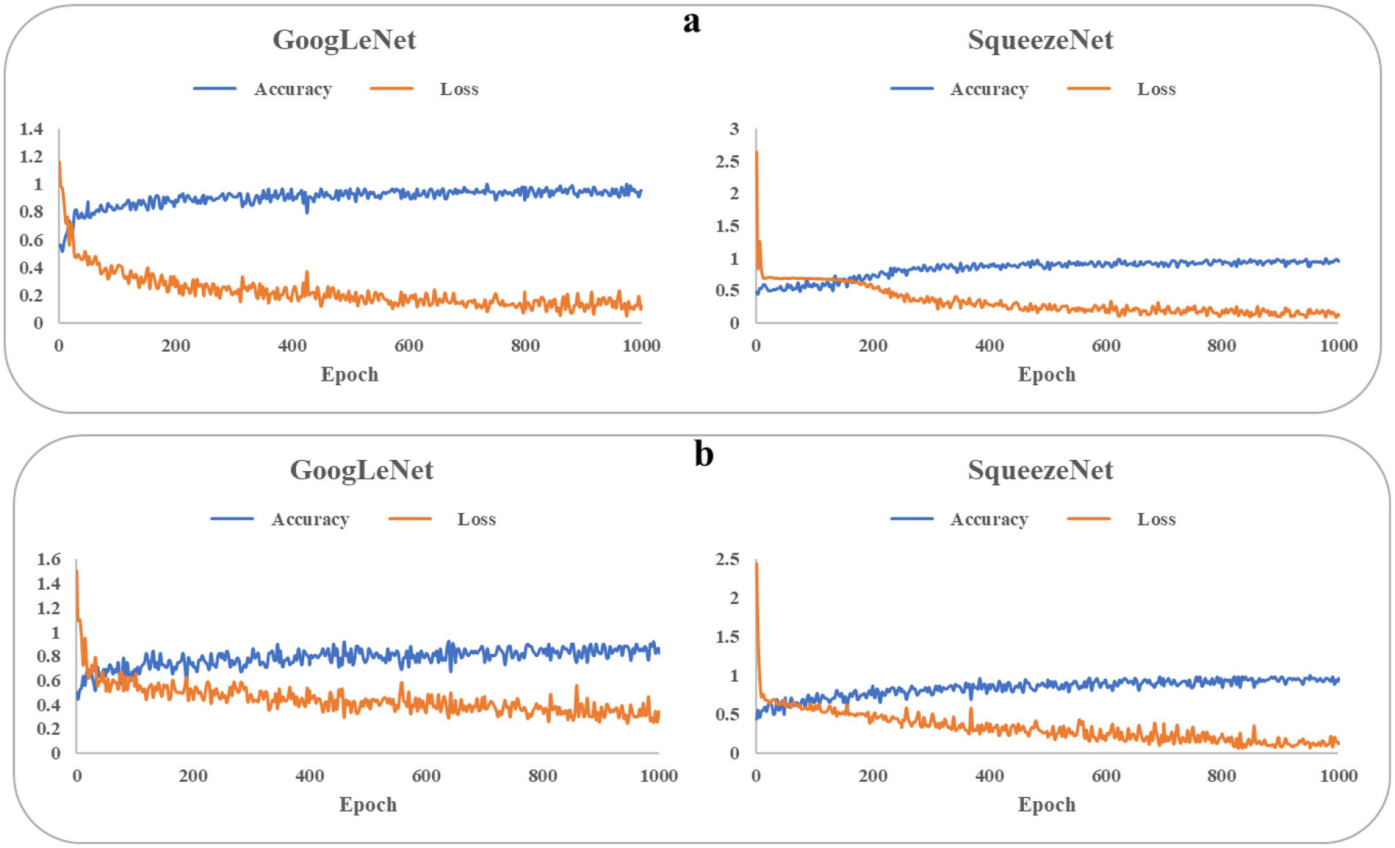
Training accuracy and loss of googLeNet and squeezeNet. baPWV, brachial-ankle pulse wave velocity. **(a)** older and young groups. **(b)** High-baPWV and low-baPWV groups.

**Figure 4:**
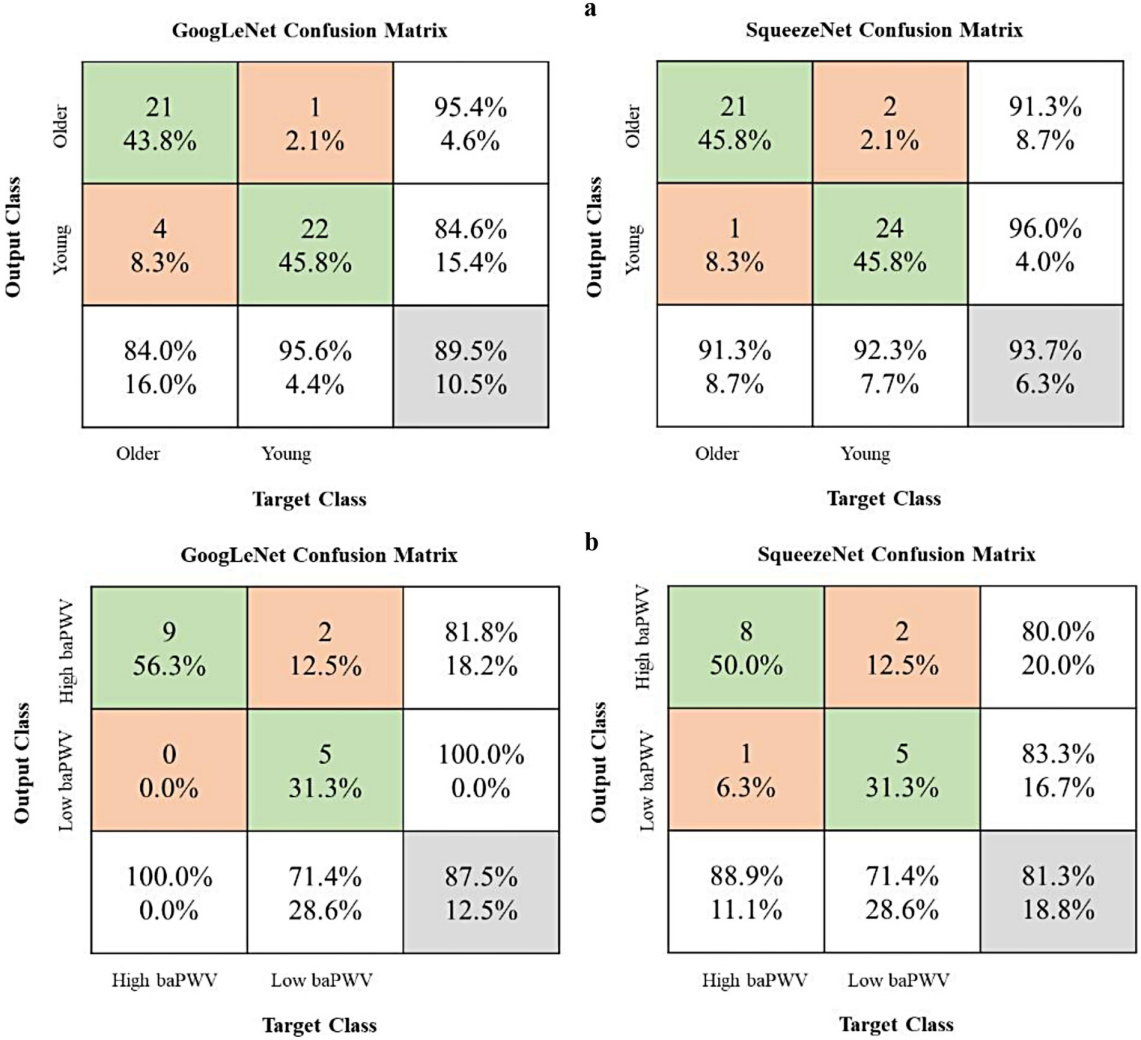
Model performance evaluation using confusion matrices. baPWV, brachial-ankle pulse wave velocity. The matrices present results for two binary classification tasks: **(a)** older group vs. young group and **(b)** high-baPWV group vs. low-baPWV group. Rows indicate the true labels, and columns indicate the predicted labels. The third row aggregates the total instances per true class and shows the Recall (Sensitivity) for each class. The third column aggregates the total instances per predicted class and shows the Precision for each class. The bottom-right cell shows the overall accuracy. Values in the main 2 × 2 cells represent counts and their percentage relative to the true class (row total).

Figure 3 illustrates the progression of training accuracy and loss for the CNN models, indicating stable learning convergence. The corresponding classification performance is quantified using confusion matrices in Figure 4. Evaluation of the age-based group classification (older vs. young) revealed that both CNN architectures showed high performance in the age-defined task (Figure 4A). GoogLeNet correctly identified 84.0% of older-group cases (sensitivity) and 95.6% of young-group cases (specificity), with an overall accuracy of 89.3%. SqueezeNet exhibited a slightly higher sensitivity of 91.3% and a specificity of 92.3%, leading to an accuracy of 93.7% (see Supplementary Material Table 6).

In the independent baPWV-defined classification task (high baPWV vs. low baPWV; Figure 4B), both models showed moderate performance (Supplementary Table 7). While this suggests that the networks capture stiffness-related information not restricted to the age-defined labels, performance was lower than in the age-defined task and should be interpreted cautiously given the limited sample size and potential spectrum differences between cohorts.

## Discussion

The assessment of arterial stiffness is closely linked to cardiovascular risk and is widely used to characterize vascular ageing and subclinical organ damage (17, 41, 42). However, the complexity, cost, and need for specialized operators limit widespread implementation of PWV, particularly in primary care workflows and in-home monitoring. This study therefore investigated the feasibility of extracting stiffness-related information from Korotkoff sounds recorded during routine cuff-based blood pressure measurement using engineered feature analysis and machine learning.

This study provides proof-of-concept evidence that Korotkoff sound analysis can differentiate between young and older individuals, groups expected to differ in vascular ageing and arterial stiffness based on the well-established association between age and PWV (43–45). Importantly, PWV was not directly measured in these two groups; therefore, these findings should be interpreted as discrimination of age-defined vascular characteristics rather than direct stiffness quantification or clinical risk stratification. In the separate hospital cohort with measured baPWV, selected features showed statistically significant associations with baPWV, providing supportive evidence for feasibility.

From a biological perspective, the link between vascular ageing and arterial stiffening is driven by integrated molecular and cellular processes across the vascular wall. A geroscience framework emphasizes that endothelial dysfunction (including impaired nitric oxide bioavailability), increased oxidative stress, chronic low-grade inflammation (“inflammaging”), mitochondrial dysfunction, impaired proteostasis, and the accumulation of senescent vascular cells with a senescence-associated secretory phenotype (SASP) contribute to progressive adverse vascular remodeling. In parallel, extracellular matrix changes, such as elastin fragmentation, increased collagen content and cross-linking, altered mechanotransduction, and, in some contexts, medial calcification, reduce arterial compliance and increase PWV. These processes provide a biologically plausible basis for why younger and older adults are expected to differ in vascular mechanical properties, even in the absence of overt cardiovascular disease, supporting the rationale for using age as a surrogate label in an initial feasibility comparison (46, 47).

Mechanistically, Korotkoff sounds are thought to arise from a combination of arterial wall vibration and hemodynamic phenomena during partial arterial compression, although the precise genesis remains an active area of research (19, 24). Recent experimental and modeling work has reinforced that both vessel wall mechanics and flow-related instabilities can contribute to sound generation and that the time–frequency characteristics are shaped by the coupled dynamics of pulsatile flow, arterial wall motion, cuff pressure, and vibration transmission through surrounding tissue (24). Increased arterial stiffness may alter the coupling between pulsatile flow, arterial wall vibration, and cuff-induced compression, which can plausibly shift the temporal asymmetry of the envelope and redistribute spectral energy. In this context, our observation that skewness and peak-frequency–related features differ between age-stratified groups is biologically plausible, because ageing-related arterial remodeling and reduced compliance can shift the dynamic response of the arterial wall to external compression and pulsatile flow (13, 48, 49).

Our findings are also consistent with prior efforts to derive vascular information from Korotkoff sounds. Earlier work reported age-related differences in Korotkoff sound characteristics as a proxy for vascular compliance (26), and recent studies have demonstrated that deep learning can extract clinically relevant information from Korotkoff sounds beyond conventional blood pressure estimation (25). Our approach emphasizes waveform- and time- frequency-derived acoustic descriptors combined with transfer-learning CNNs, offering an alternative pathway to extract stiffness-related signatures from routine cuff measurements.

Notably, the associations between individual Korotkoff sound features and baPWV were statistically significant but modest in magnitude. This is not unexpected because baPWV is influenced by multiple determinants including age, blood pressure, metabolic factors, and peripheral arterial properties, while Korotkoff sounds are additionally affected by cuff pressure dynamics, tissue characteristics, and sensor positioning. These differences in physiological territory and measurement determinants can attenuate correlations and reduce classification performance when labels are defined by baPWV rather than by age. In addition, the limited sample size of the baPWV cohort and potential spectrum differences between cohorts may constrain generalizability and performance. Therefore, the current results should be interpreted as feasibility evidence that Korotkoff sounds encode stiffness-related information, rather than as proof that these signals can replace standardized PWV measurement or provide substantial incremental risk stratification beyond age and blood pressure. Quantifying incremental value, head-to-head against estimated PWV derived from age and blood pressure, as well as multivariable clinical models, should be a central aim of future work (31).

To enable a more automated analysis, we applied transfer-learning CNNs (GoogLeNet and SqueezeNet) to classify Korotkoff sound time-frequency representations. Both architectures achieved high performance in distinguishing age-defined groups, outperforming traditional feature-based analysis. Considering potential implementation in mobile or portable devices (e.g., electronic sphygmomanometers), lightweight networks such as SqueezeNet may be advantageous in terms of computational efficiency. Because the primary labels were age-defined, these CNN results should be interpreted as proof-of-concept, and future studies should evaluate robustness across devices, measurement conditions, and populations.

Finally, while our findings support feasibility, establishing clinical utility will require additional validation steps. Larger cohorts spanning a wider range of vascular ageing should be studied with standardized PWV measurements, ideally cfPWV, to better characterize how Korotkoff sound–derived representations relate to arterial stiffness and whether they provide incremental value beyond conventional variables. Korotkoff sounds may also encode hemodynamic information beyond stiffness, but such applications remain speculative and will require dedicated study designs with appropriate labels and clinical endpoints (24).

## Limitations

Several limitations of this study should be acknowledged. First, the primary comparison (older vs. young) used age as a surrogate for arterial stiffness rather than direct PWV measurement. Although this approach is supported by prior literature and complemented by the secondary analysis in a baPWV-measured cohort, standardized direct PWV measurements within the same individuals are needed to confirm the relationship. In addition, cfPWV (reference-standard aortic stiffness) was not measured; baPWV was used as a pragmatic comparator. Future studies should therefore incorporate direct cfPWV measurements for definitive validation (17). Second, the analyzed acoustic features were selected based on *a priori* knowledge; expanding feature diversity through a more comprehensive search may yield biomarkers that better capture stiffness-related information. Third, the generalizability and model performance may be constrained by the limited sample size and cohort differences, potentially limiting further gains in accuracy and robustness. Fourth, incremental value beyond age and blood pressure was not quantified; this is required before any risk-stratification claims can be made.

### Clinical implications and future perspectives

Importantly, the current work should be interpreted as a feasibility and proof-of-concept study. The approach should be considered an adjunct rather than a substitute for established PWV assessment, and its incremental value beyond conventional variables remains to be determined. Future studies should: (i) validate performance against standardized direct PWV (preferably cfPWV) within the same individuals, (ii) evaluate test-retest reproducibility, (iii) quantify incremental value beyond conventional clinical variables, and (iv) examine associations with prospective cardiovascular outcomes to determine clinically meaningful thresholds and utility.

## Conclusion

This study supports the feasibility of using machine-learning analysis of Korotkoff sounds to capture signals related to arterial stiffness and vascular ageing. The approach discriminated age-defined groups and demonstrated a statistically significant but modest association with baPWV in a baPWV-measured clinical cohort. Further validation against standardized direct PWV measurements (ideally cfPWV) and prospective outcome studies are needed before clinical risk-stratification or prognostic claims can be made.

## Data Availability

The raw data supporting the conclusions of this article will be made available by the authors, without undue reservation.
