# Supplementary material for: Assessing arterial stiffness using characteristics of Korotkoff sounds
Source: Front Cardiovasc Med. 2026 Feb 10;13:1654162. doi: 10.3389/fcvm.2026.1654162 (PMC12930344; doi:10.3389/fcvm.2026.1654162)
Supplement: Supplementary file 1 [file Datasheet1.docx]

**Method**

**Data preprocessing**

All the data processing was performed using MATLAB (2019a). The baseline drift interference of the Korotkoff sound signal was eliminated using filtering techniques. First, the original signal is passed through a low-pass filter with a cutoff frequency of 5 Hz, and this signal is subtracted from the original signal to obtain the signal without baseline drift. In addition, the process of signal collection is affected by environmental interference; therefore, high-frequency noise must be eliminated. Wavelet transform, with its outstanding time-frequency localization ability and multi-resolution analysis characteristics, demonstrates high applicability and effectiveness in dealing with high-frequency noise, especially noise in non-stationary signals. In orthogonal wavelet transforms, the selected orthogonal basis provides a better match to the characteristics of the actual signal compared to traditional methods. This property facilitates a more effective separation of noise and other unwanted components from the data. Therefore, we chose to use a wavelet transform to remove high-frequency noise. The thresholds for 1-D wavelet denoising using the Birgé-Massart strategy are adaptive methods that can select the appropriate threshold according to the different characteristics of the signal, suppress noise and maintain useful information in the signal^1^.

**The traditional analysis method**

**Time-domain features**

As concluded by Ramakrishnan et al.^2^ , the amplitude of the Korotkoff sound signal differed between the older group and young group. In this study, the maximum amplitudes of Korotkoff sound signals in different groups were extracted. In addition, we found that the locations of the maximum amplitudes for the two group signals were different. Based on the research of Babbs et al.^3^, we speculated on the possible reasons: the vibration of the vessel wall is the main cause generating of high-frequency Korotkoff sounds (Phase Ⅱ and Ⅲ), and stiff arteries will increase the proportion of high-frequency Korotkoff sounds, thereby prolonging the third phase of Korotkoff sounds. The high-frequency sounds persist in the low-pressure area, causing the point in time at which the maximum amplitude of the Korotkoff sounds occurs to change. To simultaneously describe the amplitude and its location, and more accurately reflect the overall characteristics of the amplitude profile, we propose the concept of “center of mass”.

The concept of “center of mass” in physics refers to the unique point at the center of a mass distribution in space where the weighted position vectors relative to this point sum to zero. Similarly, in statistics, it represents the mean location of the mass distribution in space. In this study, the center of mass was defined as a weighted sum of amplitude and location information in the Korotkoff sound signals, and then averaged. Specifically, the Korotkoff sound signals were divided into frames, with the total number of frames denoted by n. Subsequently, a new axis was created with *n*/2 as the origin (*x*_1_, *x*_2_, …, *x_n_*), and the maximum amplitude of each frame signal was recorded as (*a_1_*, *a_2_*, …, *a_n_*). Finally, the amplitude of each frame signal and its corresponding new coordinate value were weighted and summed, and then divided by the sum of the amplitudes to obtain the value of the center of mass. This is expressed mathematically as Equation (1).

$$CM=\frac{a_{1}\cdot x_{1}+a_{2}\cdot x_{2}+\ldots+a_{n}\cdot x_{n}}{a_{1}+a_{2}+\ldots+a_{n}}$$

We also extracted the root mean square value, absolute mean value, kurtosis, skewness, peak factor, pulse factor, and form factor of the Korotkoff sounds. Supplementary Material Table 1 summarizes the expressions and meanings of these features.

(1)

**Frequency domain** **features**

Frequency analysis can reveal the frequency-domain​ information of the signal. The power spectrum shows how the total power of a signal is distributed as a function of frequency. In this study, we extracted the peak frequency, average frequency domain amplitude, center of gravity frequency, mean square frequency, and total power to describe the frequency distribution of Korotkoff sound signals, as shown in Supplementary Material Table 2. The peak location, center of gravity, and mean-square location were calculated along the scale axis using the scale-wise energy distribution. Therefore, the reported “peak frequency”, “center-of-gravity frequency”, and “mean-square frequency” represent scale indices (dimensionless) (or “scale units”), not Hz.

**Machine learning methods**

**CWT**

The wavelet transform provides localized information at both time and scale^4^. It decomposes the signal into a series of functions generated by a base function $\psi\left( t \right)$, named the mother wavelet. The CWT is defined as:

(2)

$$W\left( \tau,a \right)=\frac{1}{\sqrt{\left| a \right|}}\int_{-\infty}^{\infty} x\left( t \right)\cdot\Psi^{*}\left( \frac{t-\tau}{a} \right)ⅆt$$

where $x\left( t \right)$ is the signal to be analyzed, $a$ is the scale, and $\tau$ is the translation. The complex conjugate of the mother wavelet is denoted as $\Psi^{*}$.

In this study, we used the Morse wavelet, which encompasses several other analytical wavelets. It is widely employed owing to its benefits of time and frequency localization and its ability to isolate and extract quantifiable features in the time–frequency domain ^5,6^. The symmetry and time-bandwidth product parameters for this wavelet were set to 3 and 60, respectively^7-10^.

**Transfer learning**

The initial layers for extracting the low-level features were left intact, whereas the later layers for learning specific features were replaced and adjusted during training. In this study, GoogLeNet and SqueezeNet were used for transfer learning.

First, data augmentation was preformed to expand the dataset via horizontal inversion, horizontal and vertical translations (a maximum of 60 pixels), elastic deformation, and scale transformation to prevent overfitting and improve the generalizability and robustness of the model. The augmented data randomly perturbed the training data for each epoch, such that a slightly different dataset was used. Moreover, the image size was adjusted (GoogLeNet: 224×224×3, SqueezeNet: 227×227×3) to adapt to the input size of the deep learning network.

We adapted the original network and retrained it. For GoogLeNet, the last three layers ‘loss3-classifier’, ‘prob’, and ‘output’ were replaced with a fully connected layer, a SoftMax layer, and a classification output layer (2 target categories), respectively. A ‘Dropout’ layer (with a 50% random inactivation rate) was added​ before the fully connected layer to prevent overfitting. To make the new layer learn faster than the transferred layer, the learning factor of the fully connected layer was increased, and the learning rate of the weight in this layer was set to 10 times the current global learning rate. Before training, the first 80% of the backbone layer was frozen, and the freezing layer parameters were not updated during training. Iterations with the ADAM optimizer minimized the loss function. The initial learning rate, mini-batch size, and maximum number of epochs were set to 0.0001, 64, and 200, respectively. The settings for SqueezeNet were similar to those for GoogLeNet​ described above.

To evaluate the performance of the methodology proposed in this work, five indicators, namely accuracy, precision, recall, specificity and the Fl-score, were utilized.

(6)

(5)

(4)

(3)

$$Accuracy=\frac{TP+TN}{TP+TN+FP+FN}$$

$$Precision=\frac{TP}{TP+FP}$$

$$Sensitivity=Recall=\frac{TP}{TP+FN}$$

$$Specificity=\frac{TN}{TN+FP}$$

$$F1 score=\frac{2 (Precision) (Recall)}{Precision+Recall}$$

where TP (true positive) is the number of correctly predicted stiff arteries, TN (true negative) is the number of correctly predicted normal arteries, FP (false positive) is the number of incorrectly predicted stiff arteries, and FN (false negative) is the number of incorrectly predicted normal arteries. Accuracy represents the overall correctness of the event recognition. Precision and recall are particularly useful when dealing with unbalanced databases. The F1 score ranged from 0 to 1, with 1 indicating the best model output and 0 indicating the worst.

(7)

Table 1. Time domain features.

| Features | Expression | Meaning |
| --- | --- | --- |
| Root mean square | $x_{rms}=\sqrt{\frac{1}{N}\sum_{n=1}^{N} x^{2}\left( n \right)}$ | The overall intensity and distribution of the signal. |
| Absolute mean value | $\vert\bar{x}\vert=\frac{1}{N}\sum_{n=1}^{N} \vert x\left( n \right)\vert$ | The central tendency of the signal. |
| Kurtosis | $k=\frac{\sum_{n=1}^{N} {[x\left( n \right)-\bar{x}]}^{4}}{{(N-1)\sigma}^{4}}$ | The steepness and sharpness of the signal. |
| Skewness | $S=\frac{\sum_{n=1}^{N} {[x\left( n \right)-\bar{x}]}^{3}}{{(N-1)\sigma}^{3}}$ | The symmetry of the signal. |
| Peak factor | $c=\frac{max\vert x\left( n \right)\vert}{x_{rms}}$ | The magnitude of the instantaneous peak in the signal. |
| Pulse factor | $I=\frac{max\vert x\left( n \right)\vert}{\bar{x}}$ | The difference between the instantaneous peak and the mean value. |
| Form factor | $W=\frac{x_{rms}}{\bar{x}}$ | The flatness of the signal waveform |

**Note. x(n) is the signal time domain sequence, n=1,2,3, …, n; N is the sampling length;** $\boldsymbol{\sigma}$ **is the standard deviation.**

Table 2. Frequency domain features.

| Features | Expression | Meaning |
| --- | --- | --- |
| Peak frequency | $P=\frac{\left\vert s\left( k \right) \right\vert^{2}}{N}$ | The frequency with the strongest vibration |
| Average frequency domain amplitude | $S_{1}=\frac{1}{k}\sum_{k=1}^{k} s(k)$ | The average amplitude of each frequency component in the frequency domain representation |
| Center of gravity frequency | $S_{2}=\frac{\sum_{k=1}^{k} f_{k}s(k)}{\sum_{k=1}^{k} s(k)}$ | The frequency of the signal component with a larger component in the spectrum |
| Mean square frequency | $S_{3}=\frac{\sum_{k=1}^{k} {f_{k}}^{2}s(k)}{\sum_{k=1}^{k} s(k)}$ | The weighted average of the squares of the signal frequencies |
| Total power | $B=\sum_{k=1}^{k} s_{xx}\left( k \right)$ | The total energy of the signal |

**Note.** $\boldsymbol{s}\left( \boldsymbol{k} \right)$ **is the spectral sequence of the time-domain signal sequence** $\boldsymbol{x}\left( \boldsymbol{i} \right)$**,** $\boldsymbol{f}_{\boldsymbol{k}}$ **represents the frequency value of the k spectral line,** $\boldsymbol{s}_{\boldsymbol{xx}}$ **represents the power distribution of the signal in the frequency domain,** $\boldsymbol{s}_{\boldsymbol{xx}}\boldsymbol{=}\left| \boldsymbol{s}\left( \boldsymbol{k} \right) \right|^{\boldsymbol{2}}$**.**

Table 3. Linear regression models based on skewness.

Adjusted R² = 0.603

| Indicates | β | p-value | VIF |
| --- | --- | --- | --- |
| Skewness​ | 0.166 | 0.030 | 1.138 |
| Age (years) | 0.596 | <0.001 | 1.432 |
| Diabetes | 0.139 | 0.123 | 1.594 |
| SBP (mmHg) | 0.502 | <0.001 | 1.117 |
| BMI | 0.006 | 0.938 | 1.127 |

**Note:** B, Unstandardized Coefficient. β, Standardized Coefficient. VIF, variance inflation factor.

Table 4. Linear regression models based on center of mass.

Adjusted R² = 0.589

| Indicates | β | p-value | VIF |
| --- | --- | --- | --- |
| Center of Mass​ | -0.310​ | 0.018​ | 1.542 |
| Age (years) | 0.578 | <0.001 | 1.35 |
| Diabetes | 0.089 | 0.300 | 1.408 |
| SBP (mmHg) | 0.521 | 0.008 | 1.584 |
| BMI | 0.001 | 0.985 | 1.139 |

**Note:** B, Unstandardized Coefficient. β, Standardized Coefficient. VIF, variance inflation factor.

Table 5. Linear regression models based on peak frequency.

Adjusted R² = 0.594​

| Indicates | β | p-value | VIF |
| --- | --- | --- | --- |
| Peak frequency | -0.331​ | 0.029 | 1.23 |
| Age (years) | 0.581 | <0.001 | 1.409 |
| Diabetes | 0.095 | 0.266 | 1.417 |
| SBP (mmHg) | 0.482 | <0.001 | 1.155 |
| BMI | 0.018 | 0.817 | 1.137 |

**Note:** B, Unstandardized Coefficient. β, Standardized Coefficient. VIF, variance inflation factor.

Table 6. Performance metrics for two models (older group vs. young group).

| Model | Accuracy (%) | Sensitivity (%) | Specificity (%) | Precision (%) | F1 Score (%) |
| --- | --- | --- | --- | --- | --- |
| GoogLeNet | 89.5 | 84.0 | 95.6 | 95.4 | 89.3 |
| SqueezeNet | 93.7 | 91.3 | 92.3 | 91.3 | 91.3 |

Table 7. Performance metrics for two models (high baPWV group vs. low baPWV group). baPWV, brachial-ankle pulse wave velocity.

| Model | Accuracy (%) | Sensitivity (%) | Specificity (%) | Precision (%) | F1 Score (%) |
| --- | --- | --- | --- | --- | --- |
| GoogLeNet | 87.5 | 100 | 71.4 | 81.8 | 96.9 |
| SqueezeNet | 81.3 | 88.9 | 71.4 | 80 | 84.2 |

1. Cheng X, Zhang Z. Denoising method of heart sound signals based on self-construct heart sound wavelet. *AIP Advances*. 2014;4(8)doi:10.1063/1.4891822

2. Ramakrishnan D. Using Korotkoff Sounds to Detect the Degree of Vascular Compliance in Different Age Groups. *J Clin Diagn Res*. Feb 2016;10(2):CC04-7. doi:10.7860/JCDR/2016/16225.7198

3. Babbs CF. The origin of Korotkoff sounds and the accuracy of auscultatory blood pressure measurements. *J Am Soc Hypertens*. Dec 2015;9(12):935-50 e3. doi:10.1016/j.jash.2015.09.011

4. Zin ZM, Salleh SH, Daliman S, Sulaiman MD. Analysis of heart sounds based on continuous wavelet transform. presented at: Proceedings Student Conference on Research and Development, 2003 SCORED 2003; 2003;

5. Wachowiak MP, Wachowiak-Smolikova R, Johnson MJ, Hay DC, Power KE, Williams-Bell FM. Quantitative feature analysis of continuous analytic wavelet transforms of electrocardiography and electromyography. *Philos Trans A Math Phys Eng Sci*. Aug 13 2018;376(2126)doi:10.1098/rsta.2017.0250

6. Olhede SC, Walden AT. Generalized Morse wavelets. *IEEE Transactions on Signal Processing*. 2002;50(11):2661-2670. doi:10.1109/tsp.2002.804066

7. Lilly JM, Olhede SC. Generalized Morse Wavelets as a Superfamily of Analytic Wavelets. *IEEE Transactions on Signal Processing*. 2012;60(11):6036-6041. doi:10.1109/tsp.2012.2210890

8. Cartas-Rosado R, Becerra-Luna B, Martínez-Memije R, et al. Continuous wavelet transform based processing for estimating the power spectrum content of heart rate variability during hemodiafiltration. *Biomedical Signal Processing and Control*. 2020;62doi:10.1016/j.bspc.2020.102031

9. Jiang Z, Do HN, Choi J, Lee W, Baek S. A Deep Learning Approach to Predict Abdominal Aortic Aneurysm Expansion Using Longitudinal Data. *Frontiers in Physics*. 2020;7doi:10.3389/fphy.2019.00235

10. Lilly JM, Olhede SC. Higher-Order Properties of Analytic Wavelets. *IEEE Transactions on Signal Processing*. 2009;57(1):146-160. doi:10.1109/tsp.2008.2007607
